# Regular physical activity across the lifespan to build resilience against rising global temperatures

**DOI:** 10.1016/j.ebiom.2023.104793

**Published:** 2023-09-07

**Authors:** Thomas A. Deshayes, Julien D. Périard

**Affiliations:** a Montreal Heart Institute, Montréal, Canada; b School of Kinesiology and Exercise Science, Université de Montréal, Montréal, Canada; c Research Institute for Sport and Exercise, University of Canberra, Canberra, Australia

**Keywords:** Climate change, Global warming, Resilience, Adaptation, Exercise, Thermoregulation

## Abstract

Population aging, high prevalence of non-communicable diseases, physical inactivity, and rising global temperatures are some of the most pressing issues in public health of the current century. Such trends suggest that individuals increasingly less equipped to tolerate heat will be increasingly exposed to it, which from a public health perspective is alarming. Nonetheless, future impacts of extreme heat events will depend not only on the magnitude of climate change, but on our ability to adapt by becoming less sensitive and vulnerable. Although physical activity's role in mitigating climate change has received attention, its potential contribution to climate change adaptation and resilience remains largely unaddressed. Accordingly, in this viewpoint, we discuss how regular physical activity throughout life could have an important contribution to adapting to rising global temperatures, allowing to be better equipped to cope with heat-related health hazards and increasing individual and community resilience. This viewpoint constitutes a call for more research into the contribution that physical activity can have in adapting to rising global temperatures and, more broadly, to climate change.

## Introduction

Climate change,^1^ the high prevalence of non-communicable diseases (NCD),^2^^,^^3^ and alarming levels of physical inactivity^4^^,^^5^ are three central issues of the 21st century. Current and future generations will not only live in a warmer world, but experience more frequent, severe, and long-lasting extreme heat events.^1^ Trends in population aging, NCD, and physical inactivity suggest that individuals increasingly less equipped to tolerate environmental heat stress will be increasingly exposed to it. As a consequence of these issues, heat-related morbidity and mortality are expected to rise,^6^ especially among older adults and individuals with pre-existing health conditions. However, future impacts of extreme heat events will depend not only on the magnitude of climate change, but on our ability to adapt to a warmer climate by becoming less sensitive and vulnerable, and thus more resilient.^7^^,^^8^ Accordingly, there is an urgent need to move from reactive short-term solutions to long-term resilience, including better urban and building planning, improved warning systems, and a healthier population. Regarding the latter point, it has long been recognized that having good cardiorespiratory fitness helps body temperature regulation and improves heat tolerance.^9^ While this argument is not novel, its relevance for the Anthropocene Epoch and rising global temperatures resilience makes it crucial and deserving of renewed emphasis.

Recently, particular attention has been given to the role of physical activity in mitigating climate change (e.g., active mobility^10^). However, few have discussed its possible contribution to climate change adaptation and resilience. In this viewpoint, we propose that regular physical activity throughout life, which can also play an important role in achieving several United Nations Sustainable Development Goals (e.g., SDG 3: good health and well-being),^10^^,^^11^ could play an important part in adapting to rising global temperatures by increasing individual and community (e.g., healthcare systems) resilience to heat-related health hazards. In this viewpoint, the terms adaptation^d^, vulnerability^e^, and resilience^f^ are based on the definitions presented in the IPCC's AR5 and AR6 summary for Policymakers,^12^^,^^13^ but adapted specifically to the context of rising global temperatures.

Although the current viewpoint focuses primarily on the benefits of physical activity at the individual level, it is crucial to bear in mind that optimizing these benefits and those of other adaptation strategies necessitates prioritizing a synergistic approach that combines large-scale strategies (e.g., landscape/urban) with those implemented at the individual level.^14^ Large-scale urban cooling strategies that guarantee access to individuals of all age groups, abilities, and socioeconomic status to safe places for physical activity practice should be prioritized to ensure everyone's ability to engage in physical activity safely (i.e., without causing potential harm) and to maintain good health.

## The current state of health across the lifespan

The prevalence of obesity^15^^,^^16^ and NCD,^2^ such as cardiovascular diseases and type 2 diabetes mellitus, has increased rapidly in adults over the past decades and is expected to continue to rise globally. The picture is also worrying in children and adolescents, where the prevalence of obesity is increasing^15^ and an important decline in cardiovascular fitness and functional capacity has been observed for several decades.^17^, ^18^, ^19^, ^20^, ^21^ These detrimental health outcomes could drastically increase the prevalence of cardiometabolic diseases in the near future and exacerbate the pressure on healthcare systems, which is already expected to increase due to population aging.

## The role of climate change and physical inactivity in exacerbating the prevalence of health problems

Increasing evidence suggests that climate change may negatively affect human health.^22^, ^23^, ^24^, ^25^, ^26^, ^27^ Extreme heat events cause more deaths than all other natural disasters combined,^28^^,^^29^ mainly due to adverse cardiovascular events.^30^, ^31^, ^32^, ^33^ Since it is estimated that extreme heat events will be more frequent, severe and long-lasting, heat-related morbidity and mortality are expected to increase,^6^ placing even more pressure on healthcare systems.

A high prevalence of physical inactivity has already been reported, both in adolescents^5^ and adults.^4^ Worryingly, it is estimated that such a picture could worsen with climate change, as extreme weather conditions tend to dissuade people from engaging in physical activity.^34^, ^35^, ^36^ Bernard et al (2021) were the first to present a broad overview of the associations between physical activity and climate change. The expected increase in air pollution and in the frequency, severity and duration of extreme heat events and natural disasters is projected to negatively impact mid- and long-term physical activity patterns, especially in older adults, those with NCD and higher body mass index.^37^ This is concerning given that regular physical activity is an important protective factor against NCD and obesity that also helps maintaining functional and cognitive capacity, autonomy, as well as cardiorespiratory fitness throughout life.^38^, ^39^, ^40^, ^41^, ^42^ These are important key protective factors against heat-related morbidity and mortality.^8^^,^^43^, ^44^, ^45^, ^46^

## Can physical inactivity reduce adaptability and/or increase vulnerability to rising global temperatures?

Both behavioral and physiological mechanisms contribute to body temperature regulation during acute heat stress.^47^ The former depends on voluntary decisions to reduce heat exposure,^48^ and the latter is governed by a sequence of coordinated autonomic responses ranging from (i) afferent thermosensation, (ii) central integration, (iii) and efferent signaling, to (iv) thermoeffector activation (e.g., sweating and cutaneous vasodilation).^47^ It is therefore not surprising that any factor that affects these mechanisms can alter an individual's ability to respond adequately to heat stress, increasing their vulnerability. Pre-existing health conditions such as cardiovascular diseases and type 2 diabetes mellitus, taking certain medications (e.g., anticholinergics, β-blockers, antidepressants, diuretics), overweight and obesity, low functional and cognitive capacities (e.g., bed rest, low mobility, not leaving home, loss of independence, frailty), low cardiovascular fitness, and aging are associated with a reduced integrated physiological response to acute heat stress, and thus lowered heat tolerance.^8^^,^^43^^,^^45^^,^^47^^,^^49^, ^50^, ^51^, ^52^ Indeed, such conditions are associated with diminished sweating and cutaneous vasodilation, reduced cardiac function, compromised behavioral thermoregulation inherent to impaired functional and cognitive capacities, as well as impaired thermal sensation,^53^ all of which reduce the ability to dissipate heat and the capacity to meet the increased demands placed on the cardiovascular system during acute heat stress.^31^ These conditions are also associated with a lower resilience and increased risk of heat-related morbidity and mortality during extreme heat events.^8^^,^^43^^,^^45^^,^^54^, ^55^, ^56^, ^57^, ^58^ Alarmingly, the increased prevalence of health issues could create a vicious cycle, as lower cardiorespiratory fitness and functional capacity reduce the ability to engage in physical activities, which could lead to a gradual reduction of outdoor activities and exposure to different climates. In turn, this could limit the ability to heat acclimatize^59^ and thermoregulate more effectively, ultimately increasing heat vulnerability during extreme heat events.

## Can regular physical activity across the lifespan improve resilience to heat?

**Fig. 1:**
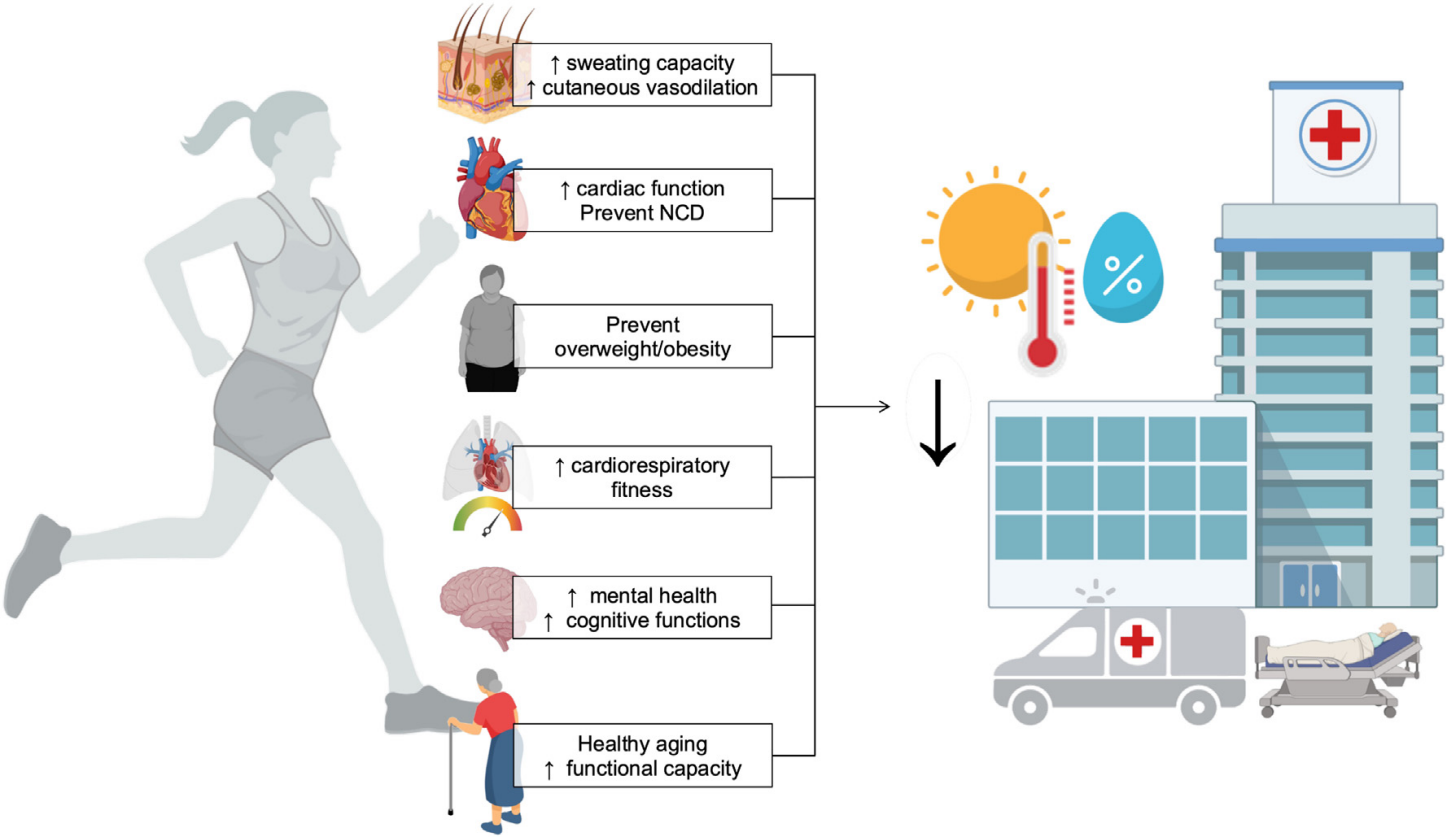
In addition to providing physiological adaptations that help to better cope with heat stress, regular physical activity is an important protective factor against non-communicable diseases (NCD) and obesity that helps maintain functional and cognitive capacity, as well as cardiorespiratory fitness throughout life. Regular physical activity is also an important contributor of mental health and healthy aging. All these factors improve heat tolerance and are key protective factors against heat-related morbidity and mortality. Created with BioRender.com.

The promotion of regular physical activity often focuses on its benefits to health and well-being and recently, on environmental issues.^10^^,^^60^, ^61^, ^62^ In addition to its potential role as a mitigation strategy (e.g., active mobility),^60^ regular physical activity could have an important contribution in adapting to rising global temperatures, allowing people to better tolerate current and future extreme heat events (Fig. 1). This, therefore, constitutes another strong argument that public health and health care professionals can consider in the benefits of adopting and maintaining an active lifestyle throughout life (i.e., multiple co-benefits).

By frequently increasing core temperature and sweating, as well as stimulating the cardiovascular system, regular physical activity (e.g., aerobic training) in temperate conditions leads to several physiological adaptations that increase heat tolerance and help in meeting the increased demands placed on the cardiovascular system during heat stress.^47^^,^^63^ However, regular physical activity while exposed to a naturally (i.e., acclimatization^59^) or artificially (i.e., acclimation^64^) hot environment provides greater and additional heat adaptations to those of aerobic training.^65^ These include a lowered resting core temperature, improved cutaneous vasodilation, increased maximal sweating capacity, improved cardiac function, expanded blood volume, and enhanced cellular protection.^64^^,^^66^ Regular physical activity also contributes to a better mental health,^67^^,^^68^ which may help cope with the stresses associated with extreme heat events.

Finally, through its important role in primary and secondary prevention, regular physical activity may help prevent the decline in cardiovascular and thermoregulatory capacity associated with obesity, certain NCD, medication, and poor cardiorespiratory fitness (Fig. 1). It may also help prevent and reduce cognitive and functional decline, allowing people to be independent and navigate easily through everyday life situations, an important factor for behavioral thermoregulation (e.g., move to a cooler space). Physical activity is also an important predictor of healthy aging. It has been suggested that age-related reductions in heat tolerance may be caused by reduced cardiorespiratory fitness, differences in body composition, and a poor state of health, rather than by advanced age *per se*.^69^ In this regard, the maintenance of regular physical activity throughout life may prevent or attenuate the rate of age-related decline in cardiovascular and sudomotor function, thereby maintaining or improving thermoregulatory capacity.^51^^,^^69^^,^^70^ This is even more important for today's children and adolescents, who will be considerably more exposed to extreme heat events than their elders were. By maintaining regular physical activity and adequate physical fitness throughout their lives, they may be better equipped to meet these challenges.^71^

However, it is important to note that several other risk factors are not directly modifiable by a healthy and active lifestyle, including, but not limited to, certain mental health disorders, low socioeconomic status, limited access to cool places or air-conditioning, among others. It is also well established that people who perform moderate to intense physical activity under hot conditions are more at risk of developing heat-related health problems (e.g., agricultural and construction workers, athletes). Hence, advocating for lifelong regular physical activity across the lifespan to build resilience against rising global temperatures must inevitably be coupled, or preempted by robust, comprehensive systemic approaches aimed at mitigating temperature increases (i.e., do no harm principle).

## How to approach physical activity in a warming world?

Given the aforementioned benefits of regular physical activity, it is essential to find solutions to continue to promote and maintain it safely, despite the projected increase in global temperature and extreme heat events. Above all, it is essential to reiterate the need to increase efforts to create favorable environments for people to practice safe physical activity as global temperatures rise (e.g., blue and green spaces, improved natural ventilation, shading infrastructures^14^). In the remainder of this section, we offer recommendations for those looking to start exercising and which can be applied by public health and health care professionals. Firstly, encourage regular physical activity, as recommended by the WHO.^72^ Briefly, for health and well-being, at least 60 min per day of moderate to vigorous (5–17 years) or 150–300 min per week of moderate (adults and older adults) aerobic physical activity are recommended, alongside reducing sedentary time.^72^ Secondly, maintaining an outdoor exercise regimen should be encouraged as the seasons change to promote heat acclimatization during summer.^59^ Avoiding exercise on unseasonably hot days during spring or early summer is also encouraged to reduce the risk of exertional heat illness.^73^ To ensure safe physical activity in hot conditions, it is suggested to reduce metabolic heat production by decreasing exercise intensity or taking more frequent breaks (in cool places if possible).^74^ A gradual approach may be preferable, starting with short duration and low intensity exercise. Other strategies can also be implemented, such as planning outdoor activities during the cooler periods of the day (e.g., early morning or evening), increasing airflow (e.g., exercising in naturally ventilated areas, using modes of exercise that increase airflow such as cycling *vs.* walking), wearing breathable clothing, avoiding direct sunlight and hot surfaces (i.e., avoid asphalt surfaces which have a lower albedo), ensuring adequate hydration, and applying cooling strategies (e.g., skin wetting, wet/cold towels).^74^ Readers are encouraged to consult the recent recommendations/policies for physical activity in hot weather for further information.^75^, ^76^, ^77^ Finally, it is advised, as frequently recommended by public health authorities, to avoid exercise during extreme heat events,^78^ or to undertake it indoors in cool spaces if possible.

## Perspectives for future studies

To pursue our understanding of the potential role of physical activity in adapting to the rise in global temperatures, we propose the following areas of future research:

1. How to continue to promote regular physical activity in a safe way for all despite more intense and frequent extreme heat events?
  1.1. Integration of physical activity into climate change adaptation policies. There is a need to assess how urban planning policies and sustainable development strategies can integrate physical activity not only in mitigating climate change, but also in promoting both human health and resilience to climate hazards. For example, how to design urban spaces conducive to physical activity that also offer protection against high temperatures or extreme weather events?
  1.2. Education and awareness campaigns to promote physical activity as a climate change adaptation strategy. Investigate the most effective communication strategies, messaging, and behavior change techniques to encourage individuals and communities to adopt physically active lifestyles in response to climate change. Assess the role of education systems, healthcare providers, and media in disseminating information and promoting behavior change. There is also an urgent need to examine methods for more effectively integrating climate change issues into the initial training and lifelong learning of diverse health professionals who advocate for physical activity promotion.
  1.3. Current environmental heat stress guidelines and limits for safe physical activity in hot weather often overlook crucial risk factors.^74^ Therefore, it is imperative to refine these guidelines to ensure the safe participation of specific populations under changing climatic conditions,^74^ particularly those at higher risk, such as older adults or individuals with cardiovascular diseases.
2. While heat acclimatization/acclimation presents an effective acute approach to adapt to rising global temperatures and extreme heat events, further research is required in understudied groups, such as the elderly and individuals with NCD, to determine if the magnitude and timeframe of acclimation is similar to younger populations.^79^ Moreover, exploring alternative heat acclimation strategies for those unable to exercise in the heat (e.g., passive exposures, water immersion) is essential.
3. The impact of regular physical activity on mental and emotional health in the context of climate change needs to be better studied. It is important to better understand how physical activity can help reduce anxiety, stress and emotional distress associated with the impacts of rising global temperatures.

## Conclusion

Through different avenues, regular physical activity across the lifespan may allow for adaptations that increase tolerance to current and future extreme heat events. Consequently, physical activity should be considered a long-term adaptative strategy to build individual and community resilience against rising global temperatures. Even if there is a great need to pursue our understanding of the potential role of physical activity in adapting to rising global temperatures, this view/perspective should be integrated into the curriculum and training objectives of public health and health care professionals.

## Contributors

T.A.D. and J.D.P. drafted the manuscript and approved the final version.

## Declaration of interests

J.D.P. has served as a consultant for The Coca Cola Company and Inuteq. No conflicts of interest, financial or otherwise, are declared by the authors.

## Footnotes

d Adapting to life in a warming climate–Processes of adjustment to actual or expected environmental temperatures and their effects on moderating harm, or taking advantage of beneficial opportunities.

e Propensity/predisposition to be adversely affected by environmental heat stress due to sensitivity or susceptibility to harm, in association with a lack of capacity to cope and adapt.

f Capacity to cope with a hazardous event (e.g., heatwave) or trend/disturbance (e.g., rising global temperatures) by responding or reorganizing in ways that maintain functioning and good health.
